# Lower Circulating Interferon-Gamma Is a Risk Factor for Lung Fibrosis in COVID-19 Patients

**DOI:** 10.3389/fimmu.2020.585647

**Published:** 2020-09-29

**Authors:** Zhong-Jie Hu, Jia Xu, Ji-Ming Yin, Li Li, Wei Hou, Li-Li Zhang, Zhen Zhou, Yi-Zhou Yu, Hong-Jun Li, Ying-Mei Feng, Rong-Hua Jin

**Affiliations:** ^1^Beijing Youan Hospital, Capital Medical University, Beijing, China; ^2^Department of Immunology, Centre for Immunotherapy, Institute of Basic Medical Sciences, Peking Union Medical College, Chinese Academy of Medical Sciences, Beijing, China; ^3^Deepwise AI Lab, Beijing, China

**Keywords:** SRAS-CoV-2, inflammation, pulmonary fibrosis, COVID-19, IFN-γ, artificial intelligence 2

## Abstract

Cytokine storm resulting from SARS-CoV-2 infection is one of the leading causes of acute respiratory distress syndrome (ARDS) and lung fibrosis. We investigated the effect of inflammatory molecules to identify any marker that is related to lung fibrosis in coronavirus disease 2019 (COVID-19). Seventy-six COVID-19 patients who were admitted to Youan Hospital between January 21 and March 20, 2020 and recovered were recruited for this study. Pulmonary fibrosis, represented as fibrotic volume on chest CT images, was computed by an artificial intelligence (AI)-assisted program. Plasma samples were collected from the participants shortly after admission, to measure the basal inflammatory molecules levels. At discharge, fibrosis was present in 46 (60.5%) patients whose plasma interferon-γ (IFN-γ) levels were twofold lower than those without fibrosis (*p* > 0.05). The multivariate-adjusted logistic regression analysis demonstrated the inverse association risk of having lung fibrosis and basal circulating IFN-γ levels with an estimate of 0.43 (*p* = 0.02). Per the 1-SD increase of basal IFN-γ level in circulation, the fibrosis volume decreased by 0.070% (*p* = 0.04) at the discharge of participants. The basal circulating IFN-γ levels were comparable with c-reactive protein in the discrimination of the occurrence of lung fibrosis among COVID-19 patients at discharge, unlike circulating IL-6 levels. In conclusion, these data indicate that decreased circulating IFN-γ is a risk factor of lung fibrosis in COVID-19.

## Introduction

Severe acute respiratory syndrome coronavirus 2 (SARS-CoV-2), which belongs to the family *Coronaviridae*, has induced the coronavirus disease 2019 (COVID-19) pandemic (1). According to the latest World Health Organization (WHO) report, the number of confirmed COVID-19-infected cases has exceeded 3 million, with more than 208,112 deaths worldwide. Clinical data from different countries have shown that approximately one third of the patients have suffered from acute respiratory distress syndrome (ARDS) (2), which is a fundamental cause of mortality and could progress to pulmonary fibrosis in survivors. In an autopsy study involving 259 patients with ARDS, the prevalence of lung fibrosis in less than 1, 1–3, or more than 3 weeks from the onset of the disease was 4, 24, and 61%, respectively (3). Consequently, a high rate of fibrosis and declined lung function were noticed in recovered COVID-19 patients (4, 5).

Structural analysis uncovered that residues in the receptor-binding domain (RBD) of SARS-CoV-2 has a high affinity to angiotensin-converting enzyme 2 (ACE2), a receptor expressed in the airway and alveolar epithelial cells (6, 7). Through ACE2-mediated endocytosis, SARS-CoV-2 endocytosed in epithelial cells are released and undergo rapid replication, leading to pyroptosis, a typical virus-linked programmed cell death (8). The release of virus RNA and damage-associated molecular patterns from dead epithelial cells further triggers inflammatory cascade in the lung, resulting in ARDS and fibrosis formation (9, 10).

Although anti-viral and anti-inflammatory drugs have been utilized for the treatment of COVID-19, the inflammatory control cascade and circumventing fibrotic lung progression for a better function are not well understood and properly defined. The objective of this study was to explore in a cross-sectional analysis the association of lung fibrosis resulting from COVID-19 with circulating immune or inflammatory molecules or both at an early stage of the disease.

## Materials and Methods

### Study Design and Participants

**FIGURE 1:**
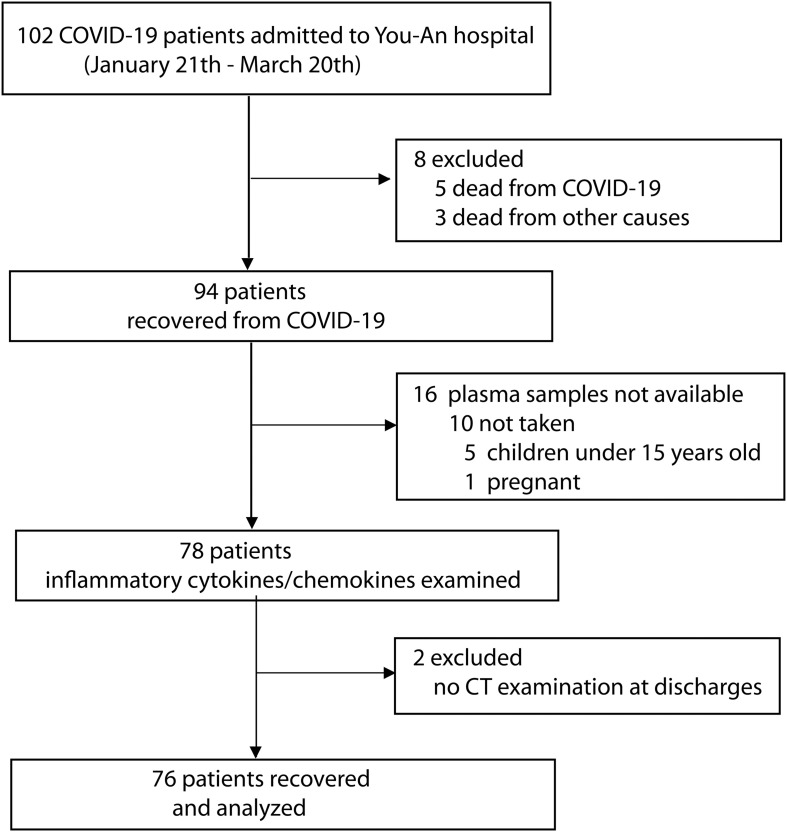
Flowchart of COVID-19 patients in the analysis.

This cohort study was performed in 102 COVID-19 patients who were admitted to Beijing Youan Hospital (designated to treat patients with SARS-CoV-2 pneumonia) between January 21 and March 20, 2020. The diagnosis of COVID-19 was based on the WHO interim guideline (11). The severity of COVID-19 was classified following the instruction of the National Institute for Viral Disease Control and Prevention, China (7th edition). Twenty-six participants were excluded from the study due to the lack of available blood samples (*n* = 17) or no CT examination (*n* = 1) or death (*n* = 8). A total of 76 patients were finally recruited, and their data were analyzed in the study. Figure 1 shows the flowchart of the study.

The epidemiological data were recorded, including local residence in Wuhan or traveling to Wuhan in the recent 14 days before the disease onset. General information such as age, practice of smoking, disease history, and drug use were collected. Previous medical history, such as disease history of cardiovascular disease, chronic respiratory disease, hypertension, and diabetes, were recorded.

The Institutional Review Board of the Capital Medical University approved the study. All participants gave written informed consent before participating in the study.

### RNA Extraction and RT-qPCR

Viral RNA was extracted from pharyngeal swabs using nucleic acids extraction kits (Lot. T124, Tianlong Science and Technology Co., Xi’an, China) on a nucleic acid extractor (GeneRotex, Tianlong Science and Technology) according to the manufacturer’s instructions. Immediately, ORF1ab and N genes of SARS-CoV-2 were detected by RT-qPCR using the SARS-CoV-2 RNA detection kit (BioGerm Medical Biotechnology Co., Ltd., Shanghai, China) on the ABI 7500 Real-Time PCR System (Thermo Fisher Scientific, Waltham, MA, United States). A Ct cut-off value was set at less than 38 for both ORF1ab and N genes for positive detection of SARS-CoV-2 virus.

### Clinical Measurements

A blood pressure of at least 140 mmHg systolic or 90 mmHg diastolic, or the use of antihypertensive drugs was recorded as being hypertensive. Individuals with plasma glucose of at least 7.0 mmol/L and fasting blood sugar of 11.0 mmol/L or more than 2 h after an orally administered glucose load of 75 g were classified as diabetics. A body temperature that was equal to or higher than 37.2°C was defined as fever.

### Biochemical Measurements

After overnight fasting, venous blood samples were obtained to measure the total and seven differential white blood cell count, creatinine, plasma glucose, and C-reactive protein (CRP). Glomerular filtration rate (eGFR) was derived from the serum creatinine by the Chronic Kidney Disease Epidemiology Collaboration (CKD-EPI) equation (12).

### Serum Biomarkers

Fasting plasma samples were collected from patients shortly after admission. The circulating levels of cytokines and chemokines were determined by a convenient bioplex kit assay (LINCO Research, Inc.) according to the manufacturer’s instruction. The inflammatory molecules included sCD40L, EGF, Eoxtaxin, FGF-2, FLT-3L, fractalkine, G-CSF, GM-CSF, GROα, IFNγ, IL-1α, IL-1β, IL-2, Il-3, IL-4, IL-5, Il-6, IL-7, IL-8, IL-9, IL-10, IL-12 (p40), IL-12 (p70), IL-13, IL-15, IL-17A, IL-17E/IL-25, IL-17F, IL-18, IL-22, IL-27, IP-10, MCP-1, MCP-3, M-CSF, MDC, MIG, MIP-1α, MIP-1β, PDGF-AA, PDGF-AB/BB, RANTES, TGFα, TNFα, TNFβ, and VEGF-A.

### Assessment of Pneumonia Characteristic by AI-Based CT Imaging System

The COVID-19 patients were examined by chest CT examination using a 256-section scanner (Philips Brilliance ICT; Dutch Philips). The CT protocol was as follows: 120 kV; automatic tube current (100–400 mA); section thickness, 5 mm; collimation, 0.625 mm; pitch, 0.914 matrix, 512 × 512; and breath-hold at full inspiration. The reconstruction kernel used was lung smooth with a thickness of 1 mm. The images were photographed at the lung (window width, 1,500 HU; window level, −500 HU) and mediastinal (window width, 350 HU; window level, 50 HU) settings. The scanning range was from the thorax entrance to the posterior costal angle.

**FIGURE 2:**
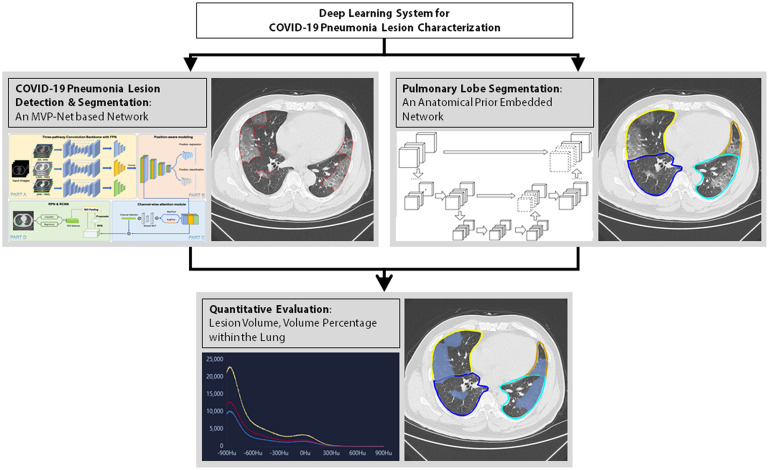
Flowchart of AI-based quantification of pulmonary fibrosis in chest CT images.

Thereafter, the index that evaluated the severity of pneumonia was computed by the AI system (Dr. Wise@Pneumonia, version 1.0, Beijing Deepwise & League of Ph.D. Technology Co., Ltd., China), which had been proved to be effective in the analysis of CT images from COVID-19 patients (13, 14). As shown in Figure 2, there were three major modules in this system. The first was the COVID-19 Pneumonia Lesion Detection and Segmentation, which aimed to detect and segment the CT image findings of COVID-19 patients. This module was achieved by the incorporation of an MVP-Net and a 3D U-Net (10), where multi-view inputs and channel-wise attention mechanism were applied followed by multiple binary classifiers. Meanwhile, the module Pulmonary Lobe Segmentation was designed to partition the pulmonary region into five pulmonary lobes, which was implemented by an anatomical prior embedded network with a smooth margin loss (15). On top of the aforementioned two modules, the Quantitative Evaluation module was capable to calculate the volume of each pneumonia lesion and its percentage compared to the volume of the entire lung. This module was very helpful to assess the severity of the fibrosis within the lung, thus leading to significant value in clinical practices (16). Data were expressed as fibrosis volume or the percentage of fibrosis within the entire lung. Figure 2 illustrates AI-based analysis of pulmonary fibrosis in the patients.

### Statistics

For the database management and statistical analysis, we used the SAS system, version 9.4 (SAS Institute Inc., Cary, NC, United States). The alanine aminotransferase (ALT), aspartate transaminase (AST), CRP, total bilirubin, serum myoglobin, creatinine kinase, creatinine, and all the circulating biomarkers were logarithmically transformed to achieve normal distribution. Normally distributed data were expressed as mean with interquartile range (IQR), while skewed data were expressed as geometric mean with IQR. Also, the proportion was expressed as *N* (%), whereas the means were compared using the large-sample *z* test and proportions by Fisher’s exact test.

Based on the fibrotic values generated by the AI-imaging analysis, the patients were categorized into two groups by the presence or absence of pulmonary fibrosis at discharge. A stepwise regression procedure was used to screen the baseline co-variables, and *p*-values for co-variables to be used in the models was set at 0.15. Therefore, the multivariate-adjusted logistical analysis was performed, and the co-variables used were age, sex, history of diabetes, and use of anti-COVID-19 treatment (antibiotics, corticosteroid, and chloroquine). When lung fibrosis was analyzed as a continuous variable, the difference of the percentage of fibrosis volume between the early stage and discharge was calculated to assess pneumonia progression for multivariate-adjusted linear regression analysis. Significance was a two-tailed *p* level of 0.05 or less.

## Results

### General Characteristics of the COVID-19 Patients

Table 1 shows the general baseline characteristics of the COVID-19 patients that were admitted and recovered in the Youan hospital. The average age was 50.5 years (range 15.1 to 84.4 years), and the percentage of women was 55.3%. Among the 76 patients, 17 (22.4%) were hypertensive. Upon admission, 63 and 13 patients were diagnosed with non-severe and severe COVID-19, respectively. Accordingly, patients with severe or critical illness were hospitalized for about 4.2 days longer than the non-severe cases [17.6 days (IQR, 15.7–19.6) vs. 13.3 days (IQR, 12.6–13.9), *p* = 0.005].

**TABLE 1:** General clinical characteristics of patients with COVID-19 at admission classified according to disease severity.

	**All patients**	**Disease severity**		***p*-value**
		**Non-severe**	**Severe**	
Number	76	63	13	
Males (%)	34 (44.7%)	26 (41.3%)	8 (61.5%)	0.18
Cardiovascular disease (%)	9 (11.8%)	6 (9.5%)	3 (23.1%)	0.15
Hypertension (%)	17 (22.4%)	11 (17.5%)	6 (46.2%)	0.02
Anti-hypertensive drugs (%)	16 (21.1%)	10 (15.9%)	6 (46.2%)	0.01
Diabetes (%)	8 (10.5%)	7 (11.1%)	1 (7.7%)	0.71
Anti-diabetic drugs (%)	8 (10.5%)	6 (9.5%)	2 (15.4%)	0.53
Transmission history (%)	37 (48.7%)	32 (50.8%)	5 (38.5%)	0.42
**Mean (IQR)**				
Age (years)	50.5 (48.4, 52.5)	48.2 (46.0, 50.4)	61.5 (57.1, 65.9)	0.004
Systolic blood pressure (mm Hg)	125.9 (123.7, 128.2)	124.8 (122.4, 127.2)	131.5 (126.0, 136.9)	0.19
Diastolic blood pressure (mm Hg)	76.1 (74.9, 77.4)	75.9 (74.6, 77.2)	77.2 (73.7, 80.7)	0.66
Respiratory symptoms				
Body temperature on admission (°C)	37.1 (37.0, 37.2)	37.0 (36.9, 37.1)	37.3 (37.1, 37.6)	0.12
White blood cells (×10^9^/L)	4.4 (4.2, 4.7)	4.3 (4.1, 4.5)	4.9 (3.8, 6.4)	0.10
Neutrophils (×10^9^/L)	2.9 (2.7, 3.1)	2.7 (2.5, 2.9)	3.5 (2.7, 4.7)	0.02
Lymphocytes (×10^9^/L)	1.1 (1.1, 1.2)	1.2 (1.1, 1.3)	0.9 (0.7, 1.1)	0.11
Monocytes (×10^9^/L)	0.37 (0.29, 0.45)	0.29 (0.27, 0.31)	0.4 (0.2, 0.5)	0.29
Platelet (×10^9^/L)	200.9 (190.3, 211.4)	201.2 (190.6, 211.9)	164.0 (146.0, 224.0)	0.94
eGFR (ml/min/1.73 m^2^)	98.7 (95.5, 102.1)	101.1 (87.2, 121.1)	79.8 (71.6, 93.4)	0.03
Saturated O_2_ (%)	96.3 (95.5, 97.1)	96.4 (95.5, 97.3)	96.1 (94.1, 98.1)	0.84
**Geometric mean (IQR)**				
ALT (U/L)	30.3 (27.9, 33.1)	30.0 (27.4, 33.1)	31.8 (26.8, 37.3)	0.79
AST (U/L)	30.9 (28.8, 33.4)	30.3 (27.4, 32.8)	35.2 (28.8, 42.9)	0.39
Total bilirubin (μmol/L)	9.3 (8.7, 10.0)	9.5 (8.8, 10.2)	8.4 (7.1, 10.0)	0.44
Serum creatinine (μmol/L)	65.3 (62.8, 68.9)	64.7 (61.5, 68.0)	69.4 (63.4, 75.9)	0.51
Myoglobin (μg/L)	44.7 (41.2, 48.4)	41.7 (38.5, 45.1)	62.8 (47.4, 83.1)	0.11
Creatinine kinase (U/L)	82.2 (74.4, 91.8)	81.4 (72.2, 91.8)	89.1 (68.0, 116.7)	0.70
C-reactive protein (mg/L)	37.7 (28.2, 49.9)	28.5 (20.9, 38.8)	109.9 (83.9,247.0)	0.01

*Data are expressed as mean (IQR), geometric mean (interquartile ranges, IQR), or *n* (%); transmission history included being resident in or traveling to Wuhan in the last 24 days. SO_2_ was measured in 40 and 13 patients in the non-severe and severe groups, respectively.*

Plasma samples were collected a few days after admission. Forty-eight inflammatory molecules level, which served as baseline profiles, were determined in the study using a magnetic bead. When the baseline levels of the inflammatory molecules were categorized by COVID-19 severity, circulating IL-5, IL-27, and VEGF-A levels were 1. 7-, 1. 5-, and 2.0-fold higher, but the MDC levels were 1.5-fold lower, in severe cases compared to non-severe cases (Supplementary Table S1).

### Evaluation of Lung Fibrosis by AI-Assisted CT Imaging

**FIGURE 3:**
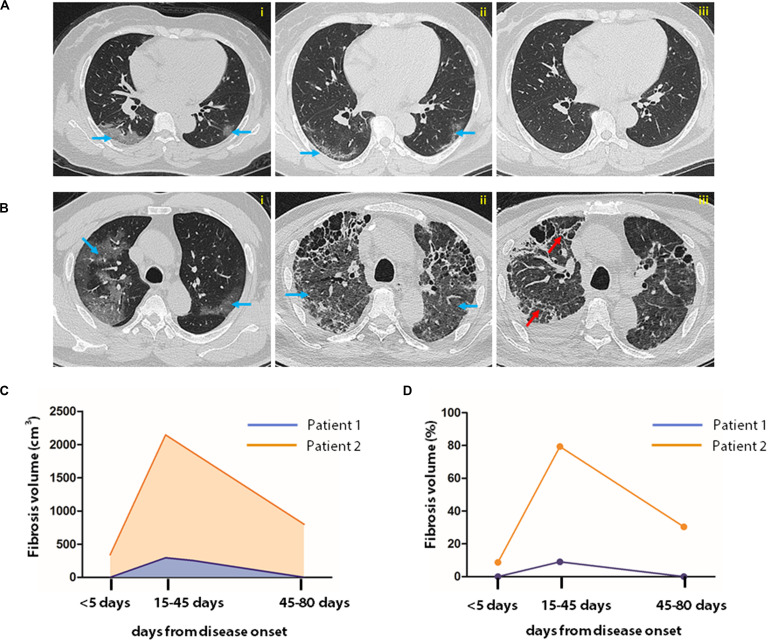
AI-assisted quantification of pneumonia lesions in COVID-19 patients. Sequential CT images of a patient with non-severe **(A)** and that of another with severe case of COVID-19 **(B)**. In the patient with severe COVID-19, CT images were obtained on the 4th, 14th, and 43rd days after disease onset **(A,i–iii)**. In patients with critical illness, CT examination was performed on the 1st, 43rd, and 80th days after disease onset **(B,i–iii)**. Fibrosis index was computed by AI system to represent fibrosis. Fibrosis volume and percentage in the entire lung are shown in images **(C,D)**. The blue and red arrows indicate typical SARS-CoV-2 infection-induced ground glass opacity and fibrosis images, respectively.

During treatment, patients underwent routine chest CT examinations. Upon discharge, two consequential tests of SARS-CoV-2 in pharyngeal swabs were negative by RT-PCR assay, and the clinical features were relieved. Observation of the CT images showed that the inflammatory signature was diminished in 30 cases, but consolidative lesion persisted in 46 cases. The representative CT images of two patients with or without fibrosis at discharge are illustrated in Figures 3A,B. To quantify further the pathological changes of pneumonia, the AI-assisted analysis of the CT images was applied to quantify fibrosis, i.e., the fibrotic volume and the percentage of fibrotic volume in the entire lung (Figures 3C,D).

### Inflammatory Profiles at Baseline Classified by Fibrosis at Discharge

Supplementary Table S2 summarizes the characteristics of COVID-19 patients at discharge by the presence or absence of lung fibrosis, identified by the AI-assisted CT imaging analysis. Concerning the COVID-19 regimen, 23 (30.3%), 15 (19.7%), and 74 (97.4%) patients received corticosteroid, chloroquine, and traditional Chinese medicine, respectively. Overall, the patients were hospitalized for an average of 14.0 days (IQR, 13.4–14.7). Patients with lung fibrosis were hospitalized 1.8 days longer than those without fibrosis (12.9 vs. 14.7 days, *p* = 0.07). The COVID-19 patients with lung fibrosis were older and had higher plasma levels of CRP and prevalence of hypertension compared with those without fibrosis (*p* < 0.001 for CRP; *p* = 0.008 for the prevalence of hypertension). By contrast, lymphocyte count and eGFR were decreased in patients with lung fibrosis compared to those without fibrosis (*p* = 0.03 for lymphocyte count; *p* < 0.001 for eGFR).

### Analysis of Lung Fibrosis as a Categorical Variable

**FIGURE 4:**
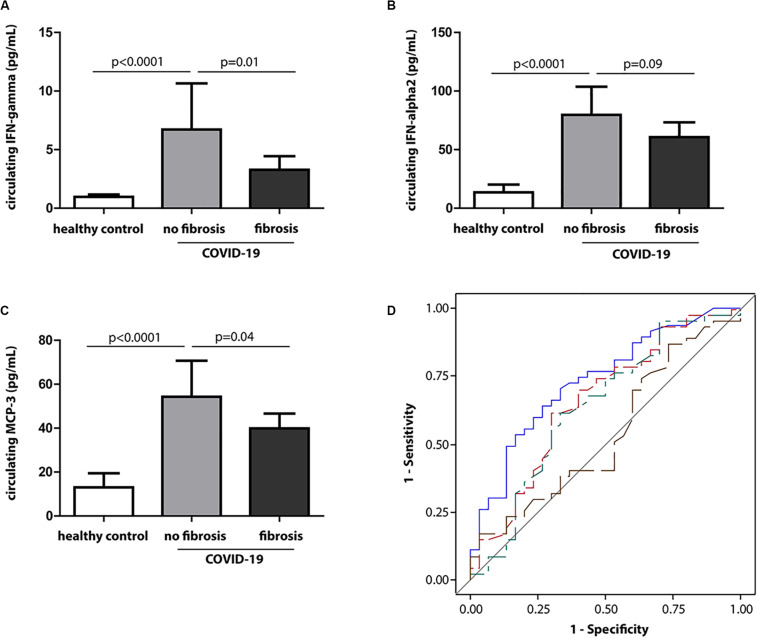
Circulating IFN-γ at baseline in relation to fibrosis at discharge. **(A–C)** Baseline levels of IFN-γ, IFN-α2, and MCP-3 in healthy controls and COVID-19 patients with the presence or absence of fibrosis at discharge. **(D)** Receiver operating characteristic (ROC) curves for discrimination of lung fibrosis (fibrotic volume >0 vs. fibrotic volume = 0) at discharge. Blue, red, green, and brown lines identify baseline CRP, MCP-3, IFN-γ, and IL-6, respectively.

The categorization of discharged COVID-19 patients by the occurrence of lung fibrosis revealed that plasma levels of interferon-γ (IFN-γ), IFN-α2, and MCP-3 were 2-, 1. 3-, and 1.3-fold lower, but the CRP was 2.6-fold higher in those with fibrosis compared to those without fibrosis (*p* = 0.01 for IFN-γ; *p* = 0.09 for IFN-α2; *p* = 0.04 for MCP-3; *p* < 0.001 for CRP) (Supplementary Tables S1, S2) (Figures 4A–C). Nevertheless, the conventional inflammatory cytokines, including IL-1β, IL-6, IL-17A, and TNFα, did not differ between the two groups (*p* ≥ 0.42 for all) (Supplementary Table S3).

Furthermore, the covariables were screened by a stepwise regression procedure. As shown in Table 2, age and the baseline levels of serum creatinine were strongly related to the incidence of fibrosis at discharge (Table 2). When adjusted by the co-founding factors, the odds ratios expressing the risk of having lung fibrosis in COVID-19 were significant for baseline IFN-γ and MCP-3, with estimates of 0.41 (95% CI, 0.20–0.86, *p* = 0.02) and 0.25 (95% CI, 0.07–0.83, *p* = 0.02), respectively (Table 3). The baseline levels of the IFN-α2 were related to reduced fibrotic events, which was not statistically significant [0.34, (95% CI, 0.10–1.13), *p* = 0.08]. However, none of the inflammatory markers measured in the study correlated with the risk of lung fibrosis at discharge (Table 3).

**TABLE 2:** Co-variables selected by stepwise regression in COVID-19 patients.

**Variables**	**COVID-19 patients**	
	**Estimate (95% CI)**	***p-*value**
Age (years)	1.16 (1.06, 1.28)	0.002
Sex (0, 1)	7.48 (0.83, 67.42)	0.07
Serum creatinine (μmol/L)	304.9 (1.7, >999.9)	0.03
History of diabetes (0, 1)	152.6 (0.7, >999.9)	0.07
Use of medications		
Anti-diabetic (0, 1)	0.01 (<0.001, 999.9)	0.10
Antibiotic (0, 1)	0.05 (0.001, 2.13)	0.12
Corticosteroid (0, 1)	19.7 (0.8, 478.1)	0.07
Chloroquine (0, 1)	0.11 (0.10, 1.17)	0.07

*p-values for entering and retaining covariables in the models were set at 0.15. Covariables considered included sex, age, mean arterial pressure, serum creatinine, alanine aminotransferase, white blood cell count, history of chronic respiratory disease, cardiovascular disease, hypertension or diabetes, use of anti-diabetic drugs, antihypertensive drugs, aspirin, and anti-COVID-19 drugs (anti-infectives, corticosteroid, chloroquine, stem cell therapy, and traditional Chinese medicine) (in class). The association sizes are expressed for estimate (SE).*

**TABLE 3:** Multivariate-adjusted associations of inflammatory cytokines at baseline and lung fibrosis at discharge.

**Biomarker (baseline)**	**Lung fibrosis vs. no fibrosis at recovery stage (46 vs. 30)**	
	**Estimate (95% CI)**	***p*-value**
Identified molecules		
IFN-γ (pg/ml)	0.41 (0.20–0.86)	0.02
IFN-α2 (pg/ml)	0.34 (0.10–1.13)	0.08
MCP-3 (pg/ml)	0.25 (0.07–0.83)	0.02
Conventional markers		
C-reactive protein (ng/ml)	1.31 (0.92–1.86)	0.14
IL-6 (pg/ml)	0.68 (0.35–1.30)	0.24
IL-1β (pg/ml)	0.85 (0.36–2.04)	0.72
TNF-α (pg/ml)	0.46 (0.10–2.01)	0.30

*The analyses were adjusted for age, sex, history of diabetes, serum creatinine, and use of anti-diabetic, antibiotics, corticosteroid, and chloroquine.*

Thereafter, we compared the sensitivity and accuracy of these molecules in association with the occurrence of fibrosis. Figure 4D demonstrates the area under the curve (AUC) for the identified molecules and conventional inflammation markers at baseline in the discrimination between the presence of fibrosis (fibrotic volume >0) vs. absence of fibrosis (fibrotic volume = 0) at discharge. Compared with CRP, the AUC was significantly lower for IL-6 (*p* = 0.007) but comparable for IFN-γ and MCP-3 (*p* ≥ 0.33).

### Analysis of Lung Fibrosis as a Continuous Variable

To further investigate whether the baseline levels of the inflammatory molecules were related to the progression of fibrosis, the difference between the percentage of fibrotic volume between discharge and admission was calculated. The univariate analysis revealed that circulating CD40L, FLT-3L, IFN-γ, IFN-α2, IL-5, IL-27, MCSF, PDGF-AA, PDGF-AA/AB, and VEGF were significantly and inversely associated with the change of fibrotic volume in percentage (Table 4). Apart from that, other inflammatory molecules measured, together with CRP, were not associated with a change of the fibrotic volume (*p* ≥ 0.06 for all). When adjusting for covariables, per 1-SD increase, fibrotic volume decreased 0.070% for IFN-γ, 0.077% for IL-5, 0.075% for PDGF-AA, 0.091% for PDGF-AA/AB, and 0.087% for VEGF, respectively (Table 4).

**TABLE 4:** Multivariate-adjusted linear association of change of fibrotic volume with inflammatory molecules at baseline.

**Biomarker (baseline)**	**Univariate association**		**Multivariate association**	
	***R***	***p-*value**	**Estimate (95% CI)**	***p-*value**
IFN-γ (pg/ml)	−0.25	0.03	−0.070 (−0.139, −0.001)	0.04
CD40L (mg/ml)	−0.24	0.04	−0.035 (−0.105, 0.034)	0.11
FLT-3L (pg/ml)	−0.10	0.02	−0.052 (−0.128, 0.024)	0.18
IFN-α2 (pg/ml)	−0.27	0.02	−0.051 (−0.120, 0.018)	0.15
IL5 (pg/ml)	−0.33	0.004	−0.077 (−0.144, −0.010)	0.03
IL27 (pg/ml)	−0.30	0.009	−0.069 (−0.141, 0.004)	0.06
MCSF (pg/ml)	−0.23	0.04	−0.047 (−0.115, 0.022)	0.20
PDGF-AA (pg/ml)	−0.27	0.02	−0.075 (−0.141, −0.009)	0.03
PDGF-AA/AB (pg/ml)	−0.32	0.005	−0.091 (−0.162, −0.020)	0.01
VEGF (pg/ml)	−0.30	0.008	−0.087 (−0.151, −0.023)	0.01

*The analyses were adjusted for antibiotics, corticosteroid, stem cell therapy, and chloroquine.*

## Discussion

To the best of our knowledge, this study is the first to evaluate the association of inflammatory molecules and pulmonary fibrosis in COVID-19 patients. The main findings of the study include (1) multivariate-adjusted logistic analysis demonstrated that the odds of having a risk of lung fibrosis at discharge were decreased with higher baseline levels of IFN-γ and MCP-3, measured in the early stage of the disease; (2) per 1-SD increase at baseline, the fibrotic volume decreased by 0.070% (95% CI, 0.001–0.139) for IFN-γ; and (3) the basal levels of IFN-γ and MCP-3 were comparable with the CRP in the discrimination of the occurrence of lung fibrosis in COVID-19 patients, whereas IL-6 were not.

Pulmonary fibrosis is one of the main abnormalities induced by SARS-CoV-2 infection (4). The SARS-CoV-2 is an enveloped RNA virus closely related to SARS-CoV and MERS-CoV, causing severe symptoms of pneumonia (1). The virus is transmitted through respiratory droplets, close contact, and other means, and asymptomatic patients could potentially transmit the virus to others unknowingly (17). Based on current observations, SARS-CoV-2 is weaker than SARS in pathogenesis but has a more robust transmission competence. Its entrance into the target cells depends on the ACE2 receptor and the serine protease TMPRSS2 for the S protein priming, both of which are highly expressed in the respiratory epithelial cells (18). Following infection, the infected cells promote the secretion of large amounts of chemokines and cytokines, leading to cytokine storm. The cytokine storm can cause lung epithelial cells and microvascular endothelial cell injury, ischemia, and hypoxia, which caused inflammatory-induced lung injury, severe pneumonia, and ARDS (19). If the cytokine storm is not timely cleared, the lung tissue will be damaged, resulting in pulmonary fibrosis.

Insight into the inflammatory profiles shows that IFN-γ, together with IL-6 and IL-10, increased in patients with a severe type of COVID-19 compared to those with a mild type (20). By contrast, another study involving 21 patients reported that IFN-γ was lower in severe COVID-19 patients compared to those with a moderate form of the disease (21). In our study, we did not observe any difference in the IFN-γ levels between moderate and severe COVID-19. Except for significant increase of CRP, IL-5, IL-27, and VEGF and decrease of MDC, the well-known conventional biomarkers such as TNFα (22), IP-10 (23), IL-6 (24, 25), and IL-1β (24) did not differ significantly between severe and non-severe cases (Supplementary Table S1). We speculate that the inflammatory profiles in the study reflected the early stage of COVID-19 before the cytokine storm happened.

TGFβ is an anti-inflammatory cytokine, which is an important mediator for acute lung injury. Its pathway is the major target for anti-fibrotic therapies (26). In our research, we have designed to see the relation between pulmonary fibrosis and pro-inflammation cytokines in COVID-19 patients. As TGF-beta is an anti-inflammatory cytokine, we have not detected the level between TGF-beta and lung fibrosis. The relationship between TGF-beta and pulmonary fibrosis in COVID-19 patients will be further investigate.

Remarkably, our findings revealed the inverse relationship of basal IFN-γ levels and lung fibrosis at discharge in COVID-19 patients. The IFN-γ is produced by lymphocytes, which are activated by specific antigens or mitogens, especially T cells and NK cells. The IFN-γ signaling plays a role in diverse cellular programs, including promoting macrophage activation and mediating host defense against pathogen infection. For example, binding of IFN-γ to the receptor activates JAK/STAT1 signaling pathways, leading to major histocompatibility complex (MHC) class I antigen processing and presentation (27). As the main drug in the treatment of the hepatitis B virus, IFN-γ treatment increases the cytotoxic T lymphocytes (CTL) epitope presentation from viral protein to elicit an immune response for virus clearance. Besides, IFN-γ induces the expression of the proteasome maturation protein (POMP), which promotes proteasome biogenesis for a more efficient antigen presentation (27).

In addition to its anti-viral activity, IFN-γ has anti-fibrotic property. The protective role of IFN-γ in kidney fibrosis was reported in IFN-γ deficient mice or in mice treated with IFN-γ blocking antibody (28). Azuma et al. showed that IFN-β inhibited bleomycin-induced lung fibrosis by decreasing the transforming growth factorβ and thrombospondin in mice (29). In patients with idiopathic pulmonary fibrosis, IFN-γ administration via inhalation delivery for 80 weeks improved the total lung capacity and diffusing capacity for carbon monoxide (30). In this study, we observed that the occurrence of pulmonary fibrosis was inversely related to IFN-γ in patients infected with SARS-CoV-2. How these molecules orchestrate to inhibit pulmonary fibrosis in COVID-19 needs future investigation.

The strengths of the study are as follows. First, CT-based evaluation of the severity of pneumonia is crude and mainly based on clinical manifestation. In the study, we introduced an AI system to quantify the pathological and dynamics changes of pneumonia, such as consolidative focus. These semi-quantitative data enabled us to dissect the roles of cytokines in pneumonia. Likewise, only one patient had a history of chronic respiratory disease. We carefully excluded the effect of any preexisting chronic respiratory abnormality by introducing the disease history as a co-variable into the model. Thus, the negative association of the circulating IFN-γ with fibrosis formation was exclusively due to SARS-CoV-2 infection. Secondly, the inflammatory profiles were measured before the onset of cytokine storm as the levels of TNF-α, IL-6, IP-10, and IL-17A did not differ between non-severe and severe type of COVID-19 patients (Supplementary Table S1).

Nevertheless, our study must be interpreted with the potential limitations. The sample size is relatively small, especially with the limited number of severe cases. Second, among all analyzed, 12 patients were transferred from other hospitals because of the disease progression. Thus, we could not collect blood samples earlier from these patients. Third, it is not feasible to quantify the viral load in the peripheral blood. Therefore, we could not assess whether the negative association of the baseline IFN-γ levels with fibrosis at discharge was mediated through the virus clearance.

## Conclusion

In conclusion, SARS-CoV-2 infection elicited inflammatory response and resulted in fibrosis formation in COVID-19 patients even after the relief of clinical symptoms and negative results from RT-PCR assay of virus RNA extracted from pharyngeal swabs. The baseline levels of IFN-γ were negatively associated with the increase of fibrosis volume in COVID-19 at discharge. These data suggest that early intervention of anti-viral infection using IFN-γ could be substantial in the inhibition of fibrosis for better functional recovery.

## Data Availability Statement

All datasets presented in this study are included in the article/Supplementary Material.

## Ethics Statement

The studies involving human participants were reviewed and approved by the Ethics Committee of Beijing Youan Hospital. Written informed consent to participate in this study was provided by the participants’ legal guardian/next of kin. Written informed consent was obtained from the individual(s), and minor(s)’ legal guardian/next of kin, for the publication of any potentially identifiable images or data included in this article.

## Author Contributions

Y-MF and R-HJ designed the research. Z-JH, JX, J-MY, LL, WH, L-LZ, and H-JL performed the research and analyzed the data. Y-MF, R-HJ, Z-JH, JX, and J-MY wrote the manuscript. ZZ and Y-ZY performed AI-based analysis of pulmonary fibrosis and participated in manuscript preparation and rebuttal. All authors contributed to the article and approved the submitted version.

## Conflict of Interest

ZZ and Y-ZY were employed by Deepwise AI Lab.

The remaining authors declare that the research was conducted in the absence of any commercial or financial relationships that could be construed as a potential conflict of interest.
