# Supplementary material for: Lower Circulating Interferon-Gamma Is a Risk Factor for Lung Fibrosis in COVID-19 Patients
Source: Front Immunol. 2020 Sep 29;11:585647. doi: 10.3389/fimmu.2020.585647 (PMC7550399; doi:10.3389/fimmu.2020.585647)
Supplement: Supplementary file 1 [file Data_Sheet_1.docx]

**Supplemental material**

Lower circulating interferon-gamma is a risk factor for lung fibrosis in COVID-19 patients

**Zhong-Jie Hu^1†^, Jia Xu^2†^, Ji-Ming Yin^1†^, Li Li^1^, Wei Hou^1^, Li-Li Zhang^1^, Zhen Zhou^3^, Yi-Zhou Yu^3^, Hong-Jun Li^1^, Ying-Mei Feng^1,^** **^*^, Rong-Hua Jin^1, *^**

^1^ Beijing Youan Hospital, Capital Medical University, Beijing 100069, China

^2^ Department of Immunology & Centre for Immunotherapy, Institute of Basic Medical Sciences, Peking Union Medical College, Chinese Academy of Medical Sciences, Beijing 100005, China

^3^Deepwise AI Lab, Beijing 100080, China

^†.^ These authors contributed equally to this work.

*** Correspondence:**Ying-Mei Feng MD, PhD
[yingmeif13@sina.com](mailto:yingmeif13@sina.com)

Rong-Hua Jin MD,

[Jin_eagle@sina.com](mailto:Jin_eagle@sina.com)

# Table S1. Inflammatory cytokine profiles of COVID-19 patients at baseline classified by severity of COVID-19.

| **Type of COVID-19** | **Non-severe** | **Severe** | **P value** |
| --- | --- | --- | --- |
| Number | 63 | 13 |  |
| sCD40L (mg/mL) | 1351.9 (1163.6, 1570.6) | 1435.5 (933.8, 2206.6) | 0.79 |
| EGF (pg/mL) | 42.1 (35.5, 50.4) | 34.1 (19.7, 59.1) | 0.63 |
| Eotaxin (pg/mL) | 106.6 (95.5, 119.0) | 142.5 (116.7, 175.8) | 0.16 |
| FGF-2 (pg/mL) | 219.1 (208.4, 230.3) | 181.2 (148.3, 221.3) | 0.14 |
| FLT-3L (pg/mL) | 12.2 (11.0, 13.3) | 14.7 (11.2, 19.1) | 0.31 |
| Fractalkine (pg/mL) | 122.7 (105.6, 142.5) | 75.9 (52.4, 111.0) | 0.15 |
| G-CSF (pg/mL) | 25.3 (21.3, 30.0) | 33.4 (22.9, 48.9) | 0.42 |
| GM-CSF (pg/mL) | 5.2 (3.8, 7.1) | 8.8 (4.4, 17.8) | 0.39 |
| GRO-α (pg/mL) | 16.0 (13.7, 18.5) | 8.5 (5.2, 13.7) | 0.07 |
| IFN-α2 (pg/mL) | 68.0 (60.9, 75.2) | 68.7 (57.4, 82.2) | 0.90 |
| IFN-ϒ (pg/mL) | 4.3 (3.7, 5.2) | 4.7 (3.0, 7.4) | 0.80 |
| IL-1α (pg/mL) | 8.5 (7.3, 5.2) | 12.4 (8.3, 18.4) | 0.21 |
| IL-1β (pg/mL) | 10.7 (9.3, 12.2) | 7.2 (5.6, 9.3) | 0.17 |
| IL-1RA (pg/mL) | 25.8 (20.3, 32.8) | 70.1 (40.0, 123.9) | 0.054 |
| IL-2 (pg/mL) | 0.6 (0.5, 0.7) | 0.6 (0.4, 0.8) | 0.89 |
| IL-3 (pg/mL) | 3.2 (2.8, 3.6) | 3.3 (2.4, 4.5) | 0.85 |
| IL-4 (pg/mL) | 1.3 (1.1, 1.5) | 1.0 (0.7, 1.4) | 0.48 |
| IL-5 (pg/mL) | 4.6 (4.1, 5.3) | 7.8 (6.4, 9.6) | 0.03 |
| IL-6 (pg/mL) | 4.9(4.0, 5.9) | 8.3 (4.9, 14.3) | 0.17 |
| IL-7 (pg/mL) | 3.6 (3.2, 4.1) | 2.6 (1.8, 3.7) | 0.29 |
| IL-8 (pg/mL) | 3.1 (2.8, 3.5) | 2.8 (2.1, 3.9) | 0.72 |
| IL-9 (pg/mL) | 21.3 (20.1, 22.4) | 18.6 (15.7, 21.4) | 0.30 |
| IL-10 (pg/mL) | 9.0 (7.4, 10.0) | 16.4 (12.2, 24.5) | 0.06 |
| IL-12 (p40) (pg/mL) | 99.4 (90.0, 109.9) | 90.0 (66.7, 109.9) | 0.40 |
| IL-12 (p70) (pg/mL) | 11.0 (10.0, 13.5) | 7.4 (5.0, 11.0) | 0.17 |
| IL-13 (pg/mL) | 20.0 (18.2, 22.2) | 13.5 (10.0, 16.4) | 0.16 |
| IL-15 (pg/mL) | 14.9 (13.5, 16.4) | 12.2 (19.0, 16.4) | 0.46 |
| IL-17A (pg/mL) | 6.0 (5.5, 7.4) | 4.1 (2.7, 6.0) | 0.27 |
| IL-17E/IL-25 (pg/mL) | 601.4 (492.4, 664.7) | 492.4 (364.8, 601.4) | 0.47 |
| IL-17F (pg/mL) | 22.2 (20.1, 24.5) | 22.2 (16.4 27.1) | 0.84 |
| IL-18 (pg/mL) | 30.0 (24.5, 36.6) | 33.1 (22.2, 44.7) | 0.82 |
| IL-22 (pg/mL) | 24.5 (20.1, 27.1) | 18.2 (13.5, 27.1) | 0.41 |
| IL-27 (pg/mL) | 1803.6 (1634.7, 1996.6) | 2695.1 (1996.6, 3291.7) | 0.03 |
| IP-10 (pg/mL) | 445.6 (330.1, 544.2) | 445.6 (181.2, 1211.1) | 0.93 |
| MCP-1 (pg/mL) | 244.5 (601.4, 270.3) | 330.1 (298.7, 403.2) | 0.12 |
| MCP-3 (pg/mL) | 44.7 (40.4, 49.4) | 49.4 (40.4, 54.6) | 0.69 |
| M-CSF (pg/mL) | 109.9 (99.4, 121.4) | 163.9 (121.4, 221.3) | 0.08 |
| MDC (pg/mL) | 601.4 (544.2, 644.7) | 403.2 (330.1, 544.2) | 0.02 |
| MIG (pg/mL) | 1667.8 (1407.0, 1957.1) | 1667.8 (1032.0, 2668.3) | 0.98 |
| MIP-1α (pg/mL) | 40.0 (37.0, 42.9) | 35.9 (29.7, 43.4) | 0.56 |
| MIP-1β (pg/mL) | 56.8 (50.9, 64.0) | 75.9 (59.1, 98.4) | 0.24 |
| PDGF-AA (pg/mL) | 1651.2 (1449.9, 1899.3) | 1539.5 (1032.0, 2296.6) | 0.84 |
| PDGF-AB/BB (pg/mL) | 17658.7 (16139.0, 19321.6) | 19321.6 (16301.2, 22003.6) | 0.59 |
| RANTES (pg/mL) | 4532.9 (4355.2, 4717.9) | 4443.2 (4020.4, 4443.2) | 0.38 |
| TGFα (pg/mL) | 6.6 (5.9, 7.4) | 5.5 (4.5, 7.4) | 0.57 |
| TNFα (pg/mL) | 74.4 (68.9, 81.4) | 73.7 (60.3, 90.0) | 0.98 |
| TNFβ (pg/mL) | 8.6 (6.7, 11.0) | 10.0 (5.5, 18.2) | 0.76 |
| VEGF-A (pg/mL) | 131.6 (109.9, 159.1) | 270.3 (200.2, 403.2) | 0.047 |

Data are expressed as geometric mean (interquartile ranges, IQR).

# Table S2. General characteristics of COVID-19 patients at discharge classified according to the absence or presence of lung fibrosis.

| Clinical characteristics | All patients | No fibrosis | Fibrosis | P value |
| --- | --- | --- | --- | --- |
| Number | 76 | 30 | 46 |  |
| Males (%) | 34 (44.7%) | 15 (50.0%) | 19 (41.3%) | 0.46 |
| Chronic respiratory disease (%) | 1 (1.3%) | 0 (0.0%) | 1 (2.2%) | 0.42 |
| Cardiovascular disease (%) | 9 (11.8%) | 1 (3.3%) | 8 (17.3%) | 0.08 |
| Hypertension (%) | 17 (22.4%) | 2 (6.7%) | 15 (32.6%) | 0.008 |
| Anti-hypertensive drugs (%) | 16 (21.1%) | 2 (6.7%) | 14 (30.4%) | 0.01 |
| Diabetes (%) | 8 (10.5%) | 2 (6.7%) | 7 (15.2%) | 0.10 |
| Anti-diabetic drugs (%) | 8 (10.5%) | 2 (6.7%) | 6 (13.0%) | 0.38 |
| Transmission history (%) | 37 (48.7%) | 12 (40.0%) | 25 (54.4%) | 0.22 |
| Treatment |  |  |  |  |
| Anti-viral (%) | 9 (11.8%) | 4 (13.3%) | 5 (10.9%) | 0.74 |
| Antibiotic (%) | 9 (11.8%) | 2 (6.7%) | 7 (15.2%) | 0.26 |
| Corticosteroid (%) | 23 (30.3%) | 3 (10.0%) | 22 (43.5%) | 0.002 |
| Chloroquine (%) | 15 (19.7%) | 9 (30.0%) | 6 (13.0%) | 0.07 |
| Stem Cell therapy (%) | 9 (11.8%) | 3 (10.0%) | 6 (13.0%) | 0.69 |
| Traditional Chinese medicine (%) | 74 (97.4%) | 28 (93.3%) | 46 (100.0%) | 0.08 |
| **Mean (IQR)** |  |  |  |  |
| Age (years) | 50.5 (48.4, 52.5) | 38.9 (36.6, 41.1) | 58.0 (55.7, 60.3) | <0.001 |
| Systolic blood pressure (mm Hg) | 125.9 (123.7, 128.2) | 122.1 (118.8, 125.3) | 128.5 (125.5, 131.5) | 0.10 |
| Diastolic blood pressure (mm Hg) | 76.1 (74.9, 77.4) | 75.3 (73.7, 76.9) | 76.7 (74.9, 78.4) | 0.51 |
| Respiratory symptoms |  |  |  |  |
| Body temperature on admission (°C) | 37.1 (37.0, 37.2) | 37.1 (36.9, 37.2) | 37.1 (37.0, 37.2) | 0.85 |
| White blood cells (x10^9^/L) | 4.4 (4.2, 4.7) | 4.3 (4.0, 4.6) | 4.5 (4.2, 4.8) | 0.51 |
| Neutrophils (x10^9^/L) | 2.9 (2.7, 3.1) | 2.6 (2.3, 2.8) | 3.1 (2.8, 3.4) | 0.10 |
| Lymphocytes (x10^9^/L) | 1.1 (1.1, 1.2) | 1.3 (1.2, 1.4) | 1.0 (1.0, 1.1) | 0.03 |
| Monocytes (x10^9^/L) | 0.37 (0.29, 0.45) | 0.30 (0.28, 0.33) | 0.4 (0.3, 0.5) | 0.38 |
| Platelet (x10^9^/L) | 200.9 (190.3, 211.4) | 218.5 (198.8, 238.2) | 189.4 (177.6, 201.1) | 0.12 |
| eGFR (ml/min/1.73m^2^) | 98.7 (95.3, 102.1) | 110.7 (105.8, 115.6) | 90.9 (86.6, 95.2) | <0.001 |
| Hospital stay (days) | 14.0 (13.4, 14.7) | 12.9 (12.2, 13.7) | 14.7 (13.7,15.7) | 0.07 |
| **Geometric mean (IQR)** |  |  |  |  |
| Alanine aminotransferase (U/L) | 30.3 (20.0, 45.0) | 30.6 (25.8, 36.6) | 30.3 (27.7, 32.8) | 0.92 |
| Aspartate aminotransferase (U/L) | 30.9 (28.8, 33.4) | 29.4 (25.5, 33.1) | 32.1 (29.4, 35.5) | 0.45 |
| Total bilirubin (μmol/L) | 9.3 (8.7, 10.0) | 9.5 (8.3, 10.8) | 9.1 (8.5, 9.9) | 0.76 |
| Serum creatinine (μmol/L) | 65.3 (62.8, 68.7) | 60.9 (58.5, 64.0) | 68.7 (64.7, 73.7) | 0.08 |
| Myoglobin (μg/L) | 44.7 (40.4, 49.4) | 36.6 (33.1, 40.4) | 51.4 (46.0, 57.4) | 0.02 |
| Creatinine kinase (U/L) | 81.4 (73.7, 90.0) | 81.4 (66.7, 90.0) | 84.7 (72.9, 97.5) | 0.78 |
| C-Reactive Protein (mg/L) | 37.7 (28.2, 49.9) | 13.5 (8.2, 20.1) | 74.4 (2.7, 102.5) | <0.001 |

Data are expressed as mean (IQR) or geometric mean (IQR) or n (%). Fibrosis is defined as consolidation index greater than 0.

# Table S3. Inflammatory cytokine profiles of COVID-19 patients at baseline classified according to the fibrotic index at the discharge.

|  | No fibrosis  (Fibrotic index=0) | Fibrosis  (Fibrotic index>0) | P value |
| --- | --- | --- | --- |
| Number | 30 | 46 |  |
| sCD40L (mg/mL) | 1221.4 (556.4, 3360.0) | 1467.5 (771.6, 2817.0) | 0.47 |
| EGF (pg/mL) | 37.2 (14.4, 150.0) | 43.0 (26.8, 110.9) | 0.40 |
| Eotaxin (pg/mL) | 123.3 (73.3, 270.9) | 105.0 (57.7, 155.8) | 0.37 |
| FGF-2 (pg/mL) | 233.1 (177.5, 293.5) | 200.9 (164.6, 258.8) | 0.14 |
| FLT-3L (pg/mL) | 13.0 (8.6, 20.6) | 12.3 (7.7, 20.1) | 0.72 |
| Fractalkine (pg/mL) | 110.6 (68.5, 239.3) | 114.6 (72.6, 236.3) | 0.65 |
| G-CSF (pg/mL) | 23.3 (9.7, 53.7) | 28.7 (16.4, 49.8) | 0.44 |
| GM-CSF (pg/mL) | 3.5 (0.8, 17.3) | 7.8 (2.7, 30.1) | 0.11 |
| GRO-α (pg/mL) | 13.4 (10.1, 28.6) | 14.9 (11.4, 31.6) | 0.47 |
| IFN-α2 (pg/mL) | 79.7 (47.7, 130.4) | 61.3 (44.2, 78.9) | 0.09 |
| IFN-ϒ (pg/mL) | 6.7 (2.6, 18.4) | 3.4 (1.8, 5.6) | 0.01 |
| IL-1α (pg/mL) | 10.1 (4.0, 25.5) | 8.5 (3.8, 17.3) | 0.57 |
| IL-1β (pg/mL) | 9.8 (6.5, 17.9) | 10.1 (7.6, 17.9) | 0.81 |
| IL-1RA (pg/mL) | 25.3 (7.0, 49.5) | 34.4 (8.4, 124.0) | 0.59 |
| IL-2 (pg/mL) | 0.7 (0.3, 1.2) | 0.5 (0.3, 1.5) | 0.37 |
| IL-3 (pg/mL) | 2.6 (1.7, 6.7) | 3.7 (2.1, 5.6) | 0.26 |
| IL-4 (pg/mL) | 1.3 (0.6, 3.9) | 1.1 (0.6, 2.2) | 0.50 |
| IL-5 (pg/mL) | 4.7 (2.5, 10.9) | 5.3 (3.3, 9.7) | 0.57 |
| IL-6 (pg/mL) | 4.6 (1.8, 10.7) | 5.9 (2.2, 14.5) | 0.42 |
| IL-7 (pg/mL) | 3.4 (1.3, 5.9) | 3.4 (1.9, 6.2) | 0.98 |
| IL-8 (pg/mL) | 2.8 (2.2, 5.0) | 3.3 (2.3, 5.3) | 0.75 |
| IL-9 (pg/mL) | 14.6 (16.2, 27.5) | 17.7 (18.6, 25.2) | 0.37 |
| IL-10 (pg/mL) | 8.1 (3.1, 17.8) | 11.0 (4.4, 31.7) | 0.26 |
| IL-12 (p40) (pg/mL) | 110.7 (61.5, 164.1) | 93.9 (73.4, 156.0) | 0.54 |
| IL-12 (p70) (pg/mL) | 10.5 (6.1, 24.5) | 10.3 (5.5, 20.1) | 0.61 |
| IL-13 (pg/mL) | 21.2 (9.9, 39.1) | 17.3 (8.6, 33.6) | 0.39 |
| IL-15 (pg/mL) | 14.2 (9.7, 25.2) | 14.6 (8.4, 26.5) | 0.60 |
| IL-17A (pg/mL) | 5.3 (3.5, 11.7) | 6.3 (2.9, 14.7) | 0.49 |
| IL-17E/IL-25 (pg/mL) | 649.3 (365.7, 860.0) | 504.7 (299.1, 758.3) | 0.14 |
| IL-17F (pg/mL) | 20.2 (14.7, 28.6) | 22.6 (15.1, 29.3) | 0.75 |
| IL-18 (pg/mL) | 34.9 (19.1, 45.4) | 27.2 (18.6, 60.7) | 0.68 |
| IL-22 (pg/mL) | 25.6 (12.8, 47.6) | 20.9 (11.7, 38.9) | 0.31 |
| IL-27 (pg/mL) | 1847.1 (1272.0, 2930.0) | 1999.7 (1337.0, 3067.0) | 0.59 |
| IP-10 (pg/mL) | 684.4 (233.7, 2838.0) | 475.0 (203.5, 1908.0) | 0.46 |
| MCP-1 (pg/mL) | 262.0 (174.4, 375.6) | 267.6 (161.8, 465.2) | 0.89 |
| MCP-3 (pg/mL) | 54.3 (34.2, 89.4) | 40.5 (28.9, 56.2) | 0.04 |
| M-CSF (pg/mL) | 104.3 (56.0, 170.9) | 129.0 (76.5, 322.5) | 0.29 |
| MDC (pg/mL) | 648.7 (453.6, 883.3) | 519.2 (380.4, 769.2) | 0.10 |
| MIG (pg/mL) | 1830.2 (1003.0, 4891.0) | 1569.9 (907.2, 3804.0) | 0.68 |
| MIP-1α (pg/mL) | 41.2 (26.5, 65.4) | 38.0 (27.8, 56.3) | 0.51 |
| MIP-1β (pg/mL) | 63.1 (37.8, 94.1) | 58.2 (34.6, 86.1) | 0.45 |
| PDGF-AA (pg/mL) | 1993.1 (1070.0, 3956.0) | 1447.3 (811.4, 2333.0) | 0.09 |
| PDGF-AB/BB (pg/mL) | 17120.0 (11537.0-25158.0) | 18465.5 (12599.0-23924.0) | 0.88 |
| RANTES (pg/mL) | 4685.0 (3804.0, 5535.0) | 4359.7 (3804.0, 4945.0) | 0.23 |
| TGFα (pg/mL) | 6.3 (4.4, 10.6) | 6.6 (4.1, 10.3) | 0.62 |
| TNFα (pg/mL) | 74.7 (43.3, 115.4) | 74.4 (50.3, 112.5) | 0.98 |
| TNFβ (pg/mL) | 9.6 (3.4, 23.0) | 8.3 (1.7, 26.8) | 0.72 |
| VEGF-A (pg/mL) | 138.4 (52.3, 364.0) | 158.6 (67.3, 296.8) | 0.95 |

Data are expressed as geometric mean (interquartile ranges, IQR).
